# Protective effect of miR‐138‐5p inhibition modified human mesenchymal stem cell on ovalbumin‐induced allergic rhinitis and asthma syndrome

**DOI:** 10.1111/jcmm.16473

**Published:** 2021-05-11

**Authors:** Huaping Tang, Xiaolei Han, Tingtian Li, Yan Feng, Jie Sun

**Affiliations:** ^1^ Department of Respiratory Medicine Qingdao Municipal Hospital Qingdao China; ^2^ Health Office Qingdao Municipal Hospital Qingdao China; ^3^ Department of International Clinic Qingdao Municipal Hospital Qingdao China

**Keywords:** allergic rhinitis and asthma syndrome, mesenchymal stem cell, miR‐138‐5p, ovalbumin

## Abstract

The objective of the study is to evaluate the protective effects of human mesenchymal stem cells (hMSCs) modified with miR‐138‐5p inhibitor against the allergic rhinitis and asthma syndrome (ARAS). MiR‐138‐5p or negative control was transfected into hMSCs, and fluorescence‐activated cell sorting was used to evaluate hMSC surface markers. Quantitative real‐time PCR (qRT‐PCR) was used to evaluate miR‐138‐5p, *SIRT1*, caspase‐3, IL‐6, IL‐1β and TNF‐α levels after TNF‐α and IL‐6 stimulations. hMSCs with or without miR‐138‐5p inhibition was intranasally administered into ARAS mice (n = 10 each group), followed by monitoring sneezing and nasal rubbing events to evaluate the allergic symptoms. Histamine, ovalbumin‐specific IgE, IgG2a, IgG1 and LTC4 release were monitored in the serum and nasal lavage fluid using enzyme‐linked immunosorbent assay. Expression of *SIRT1* and HMGB1/TLR4 pathway in nasal mucosa was assessed. After miR‐138‐5p inhibitor transfection, the hMSC lineage was preserved. Binding between *SIRT1* and miR‐138‐4p was observed, and miR‐138‐5p inhibition led to upregulation of *SIRT1*. Inhibition of miR‐138‐5p led to attenuated inflammatory responses of hMSCs upon TNF‐α and IL‐6 stimulation, and allergic symptoms in mice, as well as histamine and ovalbumin‐specific IgG release. hMSCs with miR‐138‐5p inhibition showed characteristics of activated *SIRT1* and inhibited HMGB1/TLR4 pathway. Inhibition of miR‐138‐5p in hMSCs enhanced its effects in attenuating inflammatory responses and allergic reaction in the ARAS model, which is presumably regulated by *SIRT1* and the HMGB1/TLR4 pathway.

## 1 INTRODUCTION

Allergic rhinitis and asthma syndrome (ARAS) is an allergic inflammation in the upper airway and a common chronic disease worldwide. The common symptoms of AR include sneezing, itchiness, rhinorrhoea and nasal congestion, which albeit being non‐lethal, deteriorate quality of life and lead to decreased learning ability, performance and productivity. Almost 20%‐30% of the population suffer from AR, and this disease is highly undiagnosed and incorrectly treated.^1^

AR is induced by the release of immune cells, such as antigen‐presenting cells, mast cells, B cells and eosinophils, characterized by the deficiency of regulatory T cells and imbalance of T‐helper cells.^2^ Current treatments for AR include anti‐inflammatory drugs, antihistamines, antileukotrienes agents and glucocorticoid‐based agents.^3^ Unfortunately, the long‐term recovery of AR under current treatments is still suboptimal despite the alleviation of symptoms. It remains pivotal to develop new therapeutic agents that efficiently inhibit the progression of AR.

One of the putative genes regulating AR pathogenesis is silent information regulator 1 (*SIRT1*).^4^ The regulatory functions of *SIRT1* are implicated in apoptosis, cell cycle, neuronal protection, tumorigenesis, etc Manipulation of *SIRT1* has been used as an important strategy in controlling inflammatory diseases.^5^ For example, *SIRT1* was reported as a significant regulator of insulin resistance.^6^ Therefore, *SIRT1* is a viable molecular target to alleviate AR. To manipulate *SIRT1*, a number of strategies have been proposed, among which the modification of certain microRNAs (miRNAs, miRs) has been reported with exceptional effects.^7^, ^8^

Treatment with multipotent mesenchymal stem cells (MSCs)^9^ has demonstrated promising anti‐inflammatory and immunoregulatory effects.^10^ MSCs are capable of differentiating into a number of cell types and are of high translation potential for clinical applications. MSCs are found to migrate to the airway under inflammation and exert reparative function.^11^, ^12^, ^13^ A number of previous studies have demonstrated the great potential of MSCs in treating AR.^9^, ^11^, ^14^, ^15^, ^16^ To augment the therapeutic efficacy of MSCs, genetic engineering of MSCs, that is, the use of viral or non‐viral method to enhance or knock down the expression of specific genes (DNA or RNA) of MSC, is a promising approach, which could alter both the content and secretome of MSCs purposely for high or low expression of desired molecules.^17^, ^18^

Herein, the objective of the study was to develop an approach to enhance the therapeutic effects of human mesenchymal stem cells (hMSCs) in the treatment of AR by employing miR‐138‐5p inhibition in an ovalbumin (OVA)‐induced ARAS model. MiR‐138‐5p is a putative regulator of *SIRT1*, and the upregulation of miR‐138‐5p is found to be associated with a large array of human malignancies.^19^, ^20^, ^21^, ^22^ For example, the interaction between miR‐138‐5p and *SIRT1* has been previously implicated in pancreatic cancer.^19^ However, the use of miR‐138‐5p inhibition in AR has not been explored, and the combination of miR‐138‐5p inhibition and hMSC therapy can potentially exert synergistic effects to enhance the outcome of AR therapies. The effects of the engineered hMSCs in alleviating AR symptoms, reducing inflammatory factors, as well as OVA‐specific immunological factors, were evaluated. The changes in *SIRT1* and the HMGB1/TLR4 pathway were also investigated to clarify the mechanism of action employed by the engineered hMSCs.

## 2 MATERIALS AND METHODS

### 2.1 Cell culture

hMSCs were acquired from American Type Culture Collection (ATCC, PCS‐500‐012) and maintained in DMEM culture medium (Gibco) containing 1% penicillin/streptomycin and 10% foetal calf serum (Gibco). The cells were maintained in a humidified incubator at 37°C with CO_2_ at the concentration of 5%. For tumour necrosis factor alpha (TNF‐α) and interleukin (IL)‐6 stimulation, the hMSCs were plated in 96‐well plates and incubated with 20 ng/mL TNF‐α (Roche) and 0.1 ng/mL IL‐6 (Sigma) for 24 hours.

### 2.2 Transfection

MiR‐138‐5p inhibitor and the negative control (miR‐NC) were designed and synthesized by GenePharma. The miR‐138‐5p inhibitor sequences are shown as followed: 5′‐ UGGGGUAUUUGACAAACUGAUC‐3′; MiR‐NC sequences are shown as followed: 5′‐ CAGUACUUUUGUGUAGUACAA‐3′. MiR‐138‐5p and miR‐NC (5 μL/well) were transfected into cells with Lipofectamine 2000 (Invitrogen) using manufacturer's recommendations.

### 2.3 Evaluation of MSC surface markers

Fluorescence‐activated cell sorting (FACS) coupled with flow cytometry was used to evaluate CD29, CD34, CD44 and CD45 expression in hMSCs transfected with miR‐138‐5p inhibitor or miR‐NC, compared to those without transfection.

### 2.4 Bioinformatical analysis and luciferase assay

Analysis of the binding between miR‐138‐5p and the 3′‐UTR of *SIRT1* was performed using TargetScan software and the database of starBase. Wild‐type and mutant *SIRT1* 3′UTR were cloned into pGL3‐Basic luciferase reporter vector. In HEK293T cells (Shanghai SXBIO Biotech Co., Ltd), the constructed vectors were then co‐transfected with 30 nmol/L miR‐138‐5p inhibitor or miR‐NC (GenePharma). After lysing the cells with 100 μL lysis buffer, 50 μL of cell suspension was mixed with 50 μL luciferase solution (Promega), followed by measuring the Firefly luciferase intensity. The Renilla luciferase activity was used as internal control.

### 2.5 ELISA assay

The levels of histamine and OVA‐specific IgE, IgG1, LTC4 and IgG2a in both the serum and nasal lavage fluid (NLF) were analysed using the ELISA kits (Blue Gene). In brief, serially diluted samples were transferred into 96‐well plates containing primary antibodies. After washing, tetramethylbenzidine was added and terminated with H2SO4, followed by measuring absorbance using SpectraMax microplate reader at 450 nm (Molecular Device).

### 2.6 qRT‐PCR

Total RNA was extracted from serum and NLF using the RNeasy Mini Kit (Qiagen). cDNA synthesis was conducted using the PrimerScript First Strand Kit (Takara). The Miniopticon system (Biorad) was used to perform real‐time PCR. The quantification of gene expression was conducted after normalization to the expression of beta‐actin. The sequences of primers for qRT‐PCR are listed in Table S2. The miRprimer software (Version 2.0; https://sourceforge.net/projects/mirprimer) was used for designing primers of microRNA‐specific quantitative RT‐qPCR.^23^

### 2.7 Western blot

Protein extraction from mouse nasal mucosa tissues were performed using the M‐PER Protein Extraction Reagent (ThermoFisher Scientific). Then, 30 μg of protein was loaded onto 10% gel for SDS‐PAGE, followed by transferring to a PVDF membrane. 5% non‐fat milk was added to block the membrane for 2 hours. Primary antibodies were then added and incubated with the membrane overnight at 4°C. After washing with PBST for five times, secondary antibodies were added and incubated with the membrane for 1 hour. The enhanced luminol‐based chemiluminescence mechanism was used to develop the membrane and the UVP BioSpectrum Imaging system (BioSpectrum) was used to visualize the results.

### 2.8 Construction of AR mouse model

All animal handling protocols were approved by the ethical commitment of Qingdao Municipal Hospital. Balb/c mice (female, 6 weeks old, Jackson Laboratories) were randomized to three groups (n = 10 each group). The AR group received intraperitoneal injection of OVA (10 μg) and AI(OH)_3_ (2 mg) in 100 μL saline on 0, 7th and 14th day, followed by challenging with OVA solution (40 mg/mL; 20 μL/mouse) in nostrils from day 21 to 28. The NC group were normal mice that received the AI(OH)_3_ (2 mg) solution in saline at the same schedule. In the miR‐NC and miR‐138 in groups, mice were intranasally injected with 1 million hMSCs modified with miR‐NC or miR‐138‐5p inhibitor, respectively, at 30 minutes before OVA challenge from 21 to 28 days.

At day 42, the frequency of sneezing and rubbing events of all mice were recorded for every 10 minutes for a total of 1 hour. The average number of events per 10 minutes was recorded. The blood and NLF were then collected and centrifuged for 10 minutes at 1600 g. After sacrificing the mice, nasal mucosa tissues were collected.

### 2.9 Counting inflammatory cells in NLF

NLF were collected before sacrificing the mice and further diluted with 1 mL PBS. Total numbers of leucocytes, eosinophils, lymphocytes and neutrophils were manually counted using a haemocytometer (Mindray 3000) under a light microscope after Wright's‐Giemsa staining.

### 2.10 Statistical analysis

The data were presented as mean ± standard deviation (SD) and one‐way analysis of variance was used to analyse the differences. Turkey's post hoc test was used for multiple‐group comparisons. The differences with *P* < .05 were considered statistically significant.

## 3 RESULTS

### 3.1 hMSCs modified with the miR‐138‐5p inhibitor

We modified hMSCs using miR‐138‐5p inhibitor and the negative control (NC) with the aim of enhancing its protective effects against AR. First, to characterize the modified hMSCs, we used FACS to analyse the expression of hMSC surface markers, including CD29, CD44, CD34 and CD45 (Table S1 and Figure S1). The levels of these markers did not significantly differ in unmodified hMSCs (control) and hMSCs transfected with miR‐NC or the miR‐138‐5p inhibitor (n = 4, *P* > .05), and the low CD34 and CD45 expression along with the high CD28 and CD44 expression suggested the preservation of the hMSC lineage.

### 3.2 MiR‐138‐5p directly targeted SIRT1 and regulated the expression of SIRT1 in hMSCs

**FIGURE 1:**
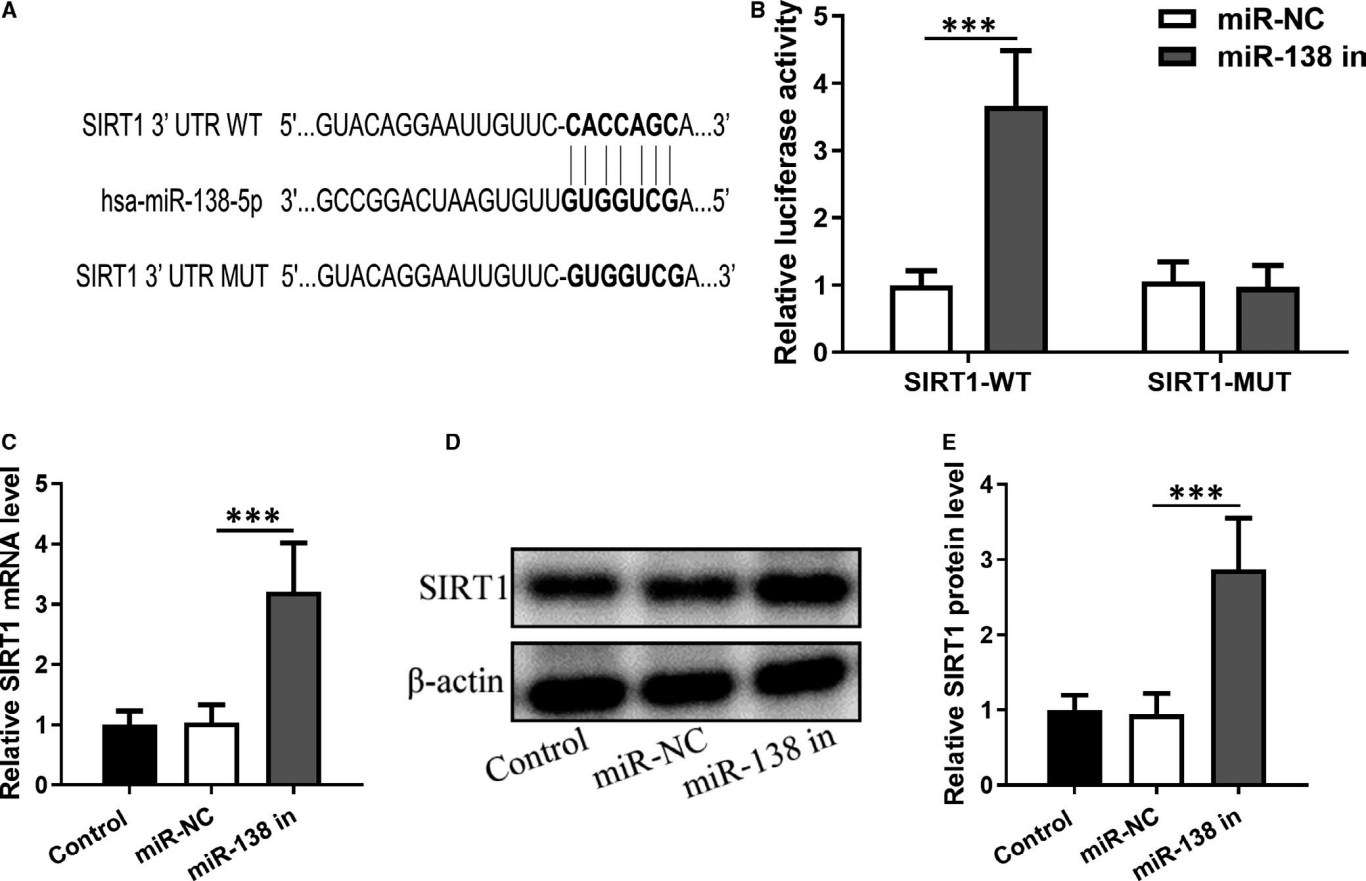
miR‐138‐5p directly binds and regulates the expression of *SIRT1*. A, The predicted binding of hsa‐miR‐138‐5p with the wild‐type 3′‐UTR region of *SIRT1*. A mutated 3′‐UTR of *SIRT1* abrogates the binding. B, Wild‐type (WT) *SIRT1* or mutant (MUT) *SIRT1* was co‐transfected with miR‐138‐5p inhibitor or miR‐NC into HEK293T cells. After 24 h, relative luciferase activities were assessed. C, qRT‐PCR measurement of relative *SIRT1* mRNA levels after 24 h of transfection. D, Western blotting analysis of SIRT1 protein levels after 24 h of transfection and relative expressions were normalized to control group (E)

Bioinformatical analysis using TargetScan (http://www.targetscan.org/vert_72/) indicated that a binding site might exist between the 3′‐UTR of *SIRT1* and miR‐138‐5p, while we constructed a mutant *SIRT1* 3′‐UTR without the binding site (Figure 1A). The interaction between wild‐type *SIRT1* and miR‐138‐5p was validated by luciferase assay, whereby transfection of miR‐138‐5p inhibitor induced an upregulation of wild‐type *SIRT1* but not the mutant *SIRT1* (Figure 1B). Concomitant with miR‐138‐5p knockdown, SIRT1 was shown to be upregulated in both mRNA (Figure 1C) and protein levels (Figure 1D‐E).

### 3.3 Inhibition of miR‐138‐5p attenuated inflammatory response in TNF‐α and IL‐6 stimulated hMSCs

**FIGURE 2:**
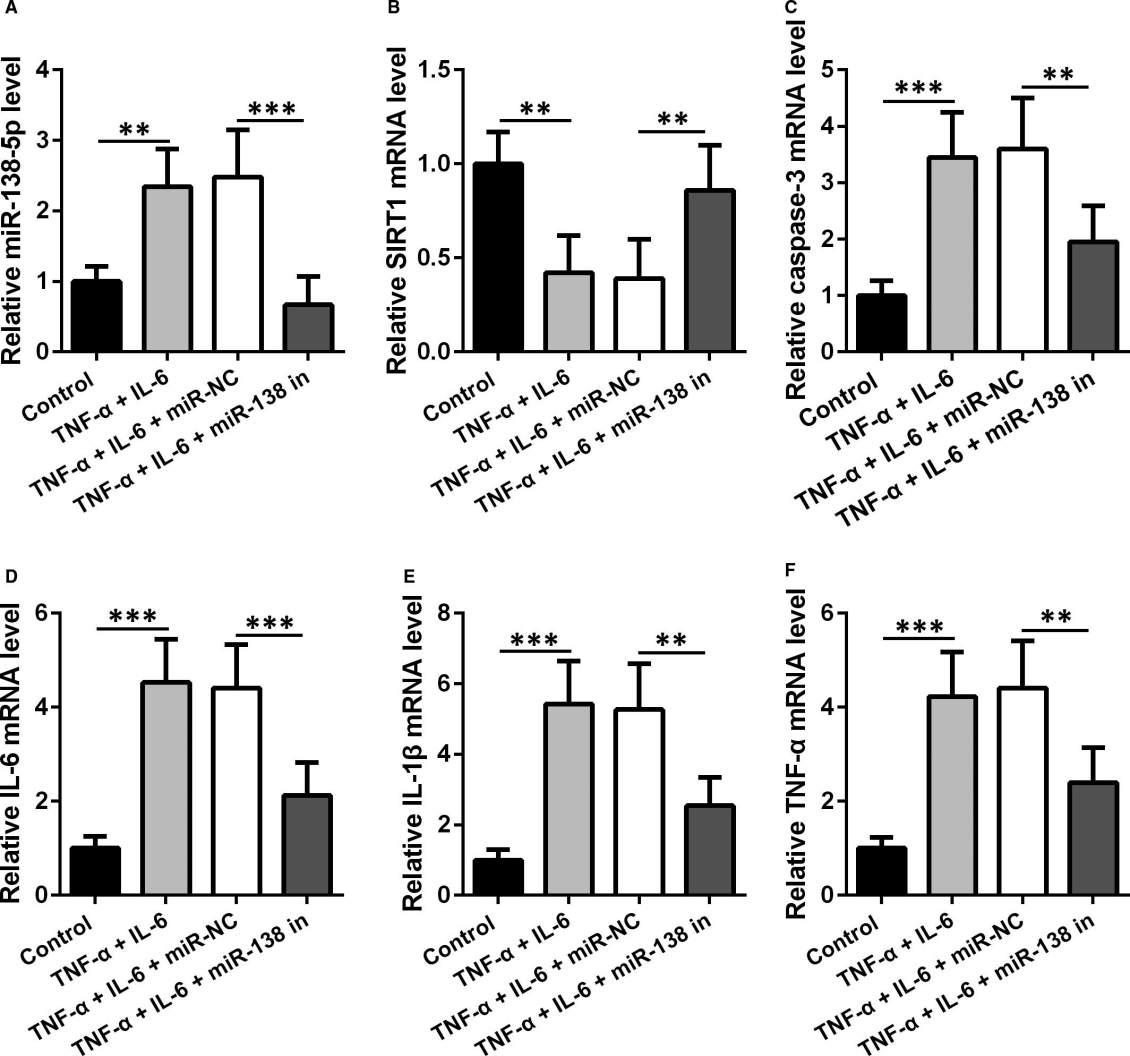
Inhibition of miR‐138‐5p regulated inflammatory response in TNF‐α and IL‐6 stimulated hMSCs. hMSCs were transfected with miR‐138‐5p inhibitor then treated with IL‐6 and TNF‐α for 24 h. (A, B) qRT‐PCR analysis of the expressions of miR‐138‐5p (A) and *SIRT1* (B) mRNA. C‐F, The expressions of caspase‐3, IL‐6, IL‐1β and TNF‐α were measured by qRT‐PCR

We next examined the effect of miR‐138‐5p inhibition on the responses of hMSCs upon TNF‐α and IL‐6 stimulation. TNF‐α and IL‐6 treatment is known to trigger inflammatory responses and apoptosis in hMSCs, characterized by upregulation of miR‐138‐5p, caspase‐3, IL‐1β, IL‐6 and TNF‐α, as well as downregulation of *SIRT1* (Figure 2A‐F). As shown in Figure 2A, stimulation with IL‐6 and TNF‐α led to significantly upregulated miR‐138‐5p levels (*P* < .001). In the presence of miR‐138‐5p inhibition, the miR‐138‐5p level, however, was not significantly changed after TNF‐α and IL‐6 stimulation, which was in contrast with the pronounced upregulation of miR‐138‐5p in the presence of miR‐NC transfection (*P* < .001). Similarly, miR‐138‐5p inhibition abrogated the changes in *SIRT1* (Figure 2B), caspase‐3 (Figure 2C), IL‐6 (Figure 2D), IL‐1β (Figure 2E) and TNF‐α (Figure 2F) induced by TNF‐α and IL‐6 treatment, suggesting the function of miR‐138‐5p inhibition in suppressing inflammatory responses and apoptosis.

### 3.4 Intranasally administered hMSCs with miR‐138‐5p inhibition attenuates allergic symptoms in AR mice

**FIGURE 3:**
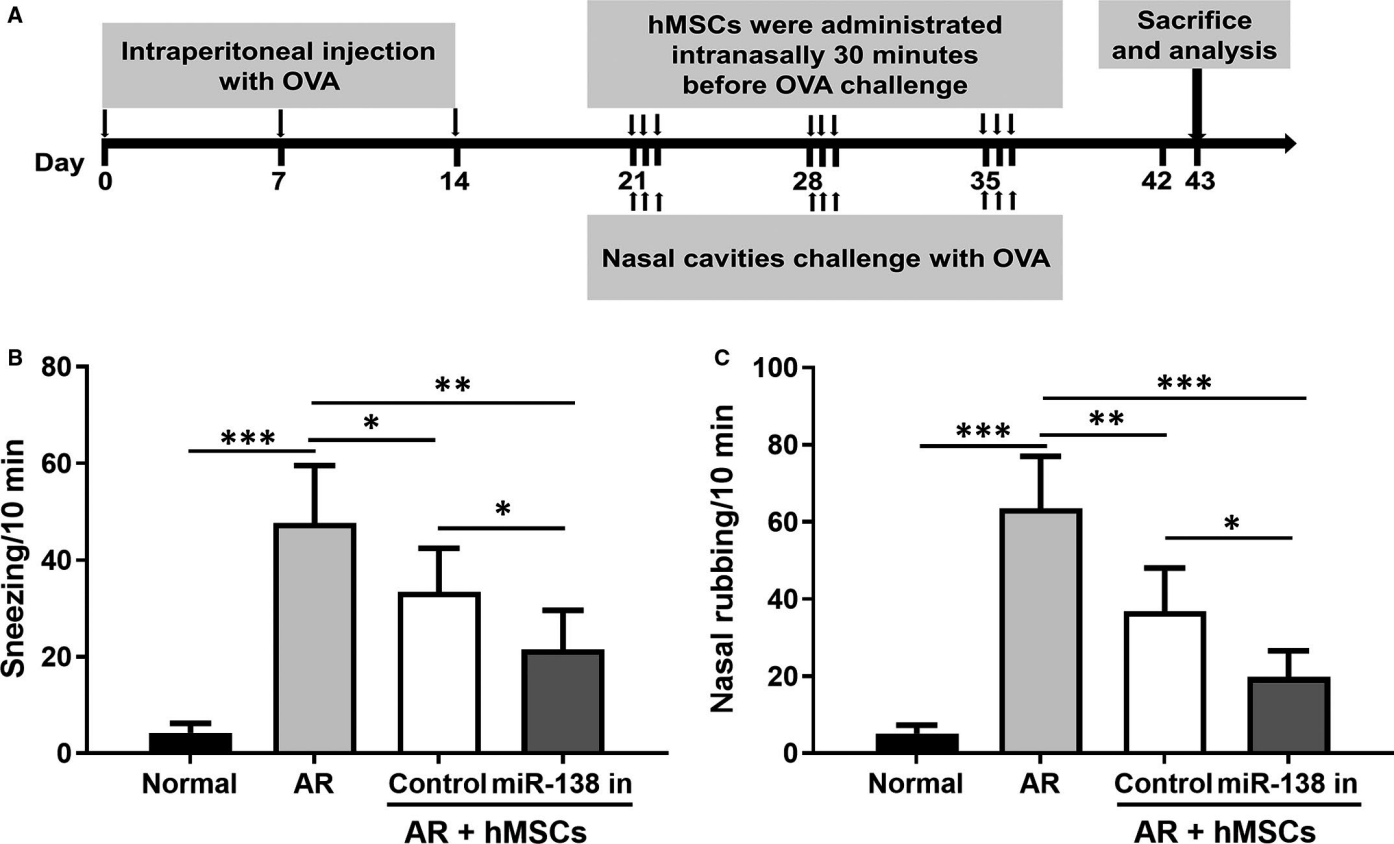
miR‐138‐5p inhibition modified hMSCs intranasal administration attenuated allergic symptoms in allergic rhinitis mice. A, Schematic diagram illustration of the construction and treatment of mouse ovalbumin‐induced allergic rhinitis mouse model. B, Number of sneezing and (C) Number of nasal rubbing events. N = 10

Next, we evaluated the protective effects of the miR‐138‐5p inhibitor engineered hMSCs in the AR model. AR mice were constructed by OVA intraperitoneal injection once a week for 2 weeks. During weeks 3‐5, mice were challenged with OVA once a week, and received hMSC treatment intranasally at 30 minutes before OVA challenge. Sneezing events and nasal rubbing events were recorded, and mice were sacrificed at day 43 (Figure 3A). While sneezing and nasal rubbing events were significantly lower in AR mice after treatment with unmodified hMSC, a more pronounced reduction in these events was seen after treatment with hMSCs with miR‐138‐5p inhibition (*P* < .05 for both sneezing and rubbing events; Figure 3B‐C).

### 3.5 hMSCs with miR‐138‐5p inhibition attenuates histamine levels

**FIGURE 4:**
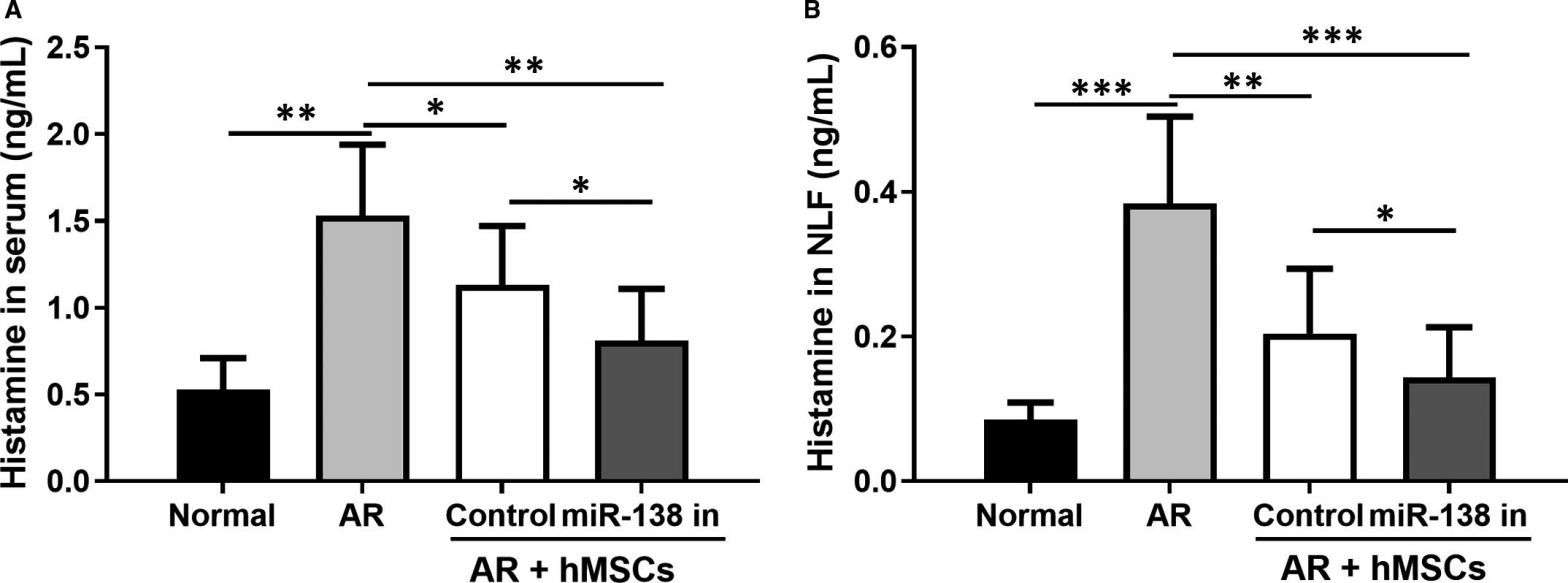
miR‐138‐5p inhibition modified hMSCs intranasal administration attenuated the release of histamine in serum (A) and nasal lavage fluid (NLF; B). N = 10

After sacrificing the mice, the serum and NLF were collected. Since histamine level is a putative indicator of allergic reaction, we measured histamine levels in the serum and NLF in all mouse groups. We showed here that in AR mice, histamine was markedly higher in the serum and NLF of AR models than normal control (*P* < .01, histamine serum level of AR group compared to NC group; *P* < .01 histamine NLF level of AR group compared to NC group). hMSC treatment significantly lowered the levels of histamine in the serum (*P* < .05 vs AR mice) and NLF (*P* < .01, vs AR mice; Figure 4A‐B). This reduction of histamine was further enhanced by hMSCs modified with miR‐138‐5p inhibition (*P* < .05 for both serum and NLF levels, vs hMSC treatment control group; Figure 4A‐B).

### 3.6 Analysis of OVA‐specific IgE, IgG1, LTC4 and IgG2a

**FIGURE 5:**
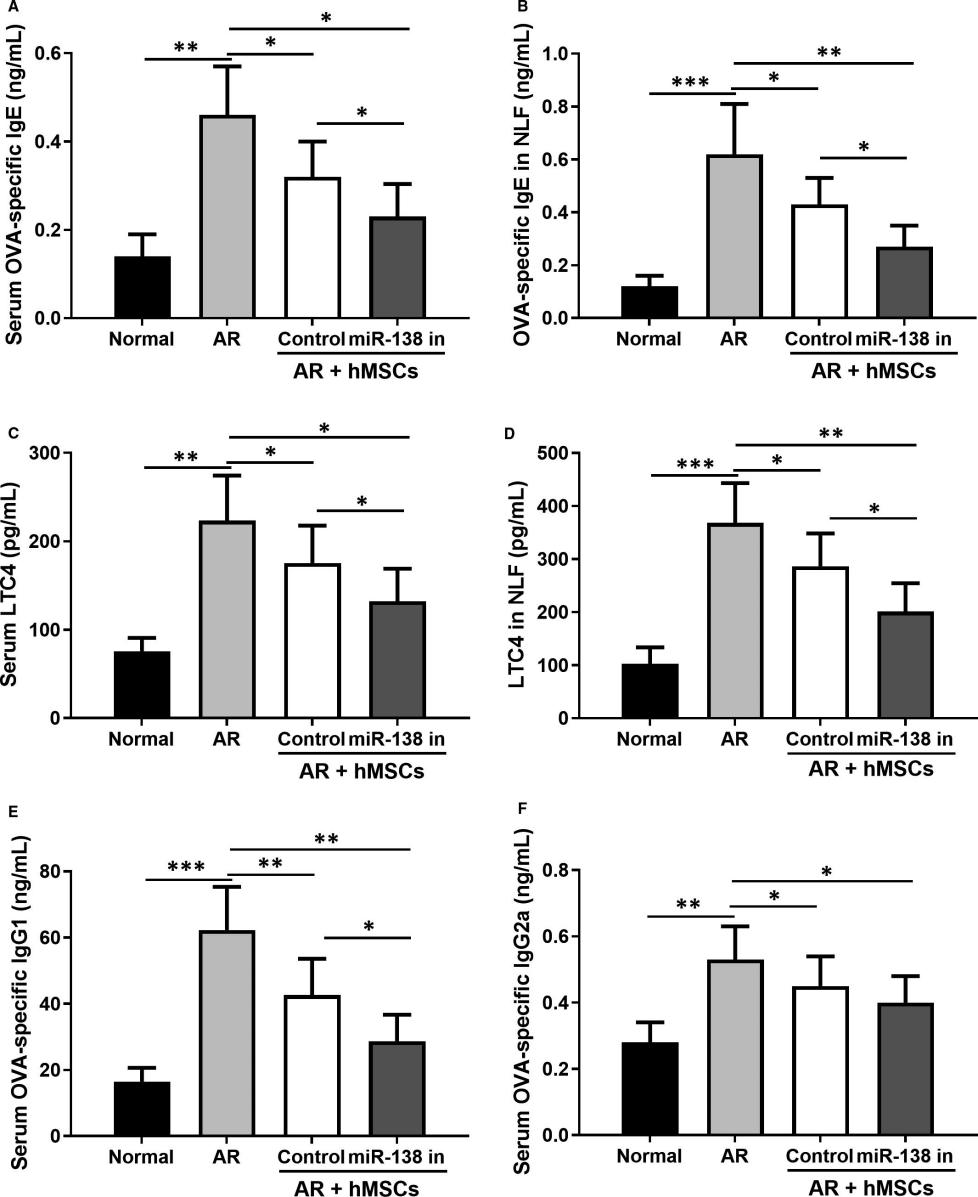
miR‐138‐5p inhibition modified hMSCs intranasal administration attenuated the OVA‐specific IgE (A and B), IgG1 (E), IgG2a (F) and LTC4 (C and D) release in serum and NLF from AR models. N = 10

In AR model mice, OVA treatment resulted in upregulation of OVA‐specific IgE, IgG1, LTC4 and IgG2a in both the NLF and serum (Figure 5A‐F). Treatment with unmodified hMSCs led to significant reduction of these factors, with treatment with hMSCs with miR‐138‐5p inhibition showing the most prominent reduction. These evidence further supported that miR‐138‐5p inhibition in hMSCs was effective in reducing OVA‐specific allergic reactions.

### 3.7 Analysis of serum inflammatory factors and inflammatory cells in the NLF

**FIGURE 6:**
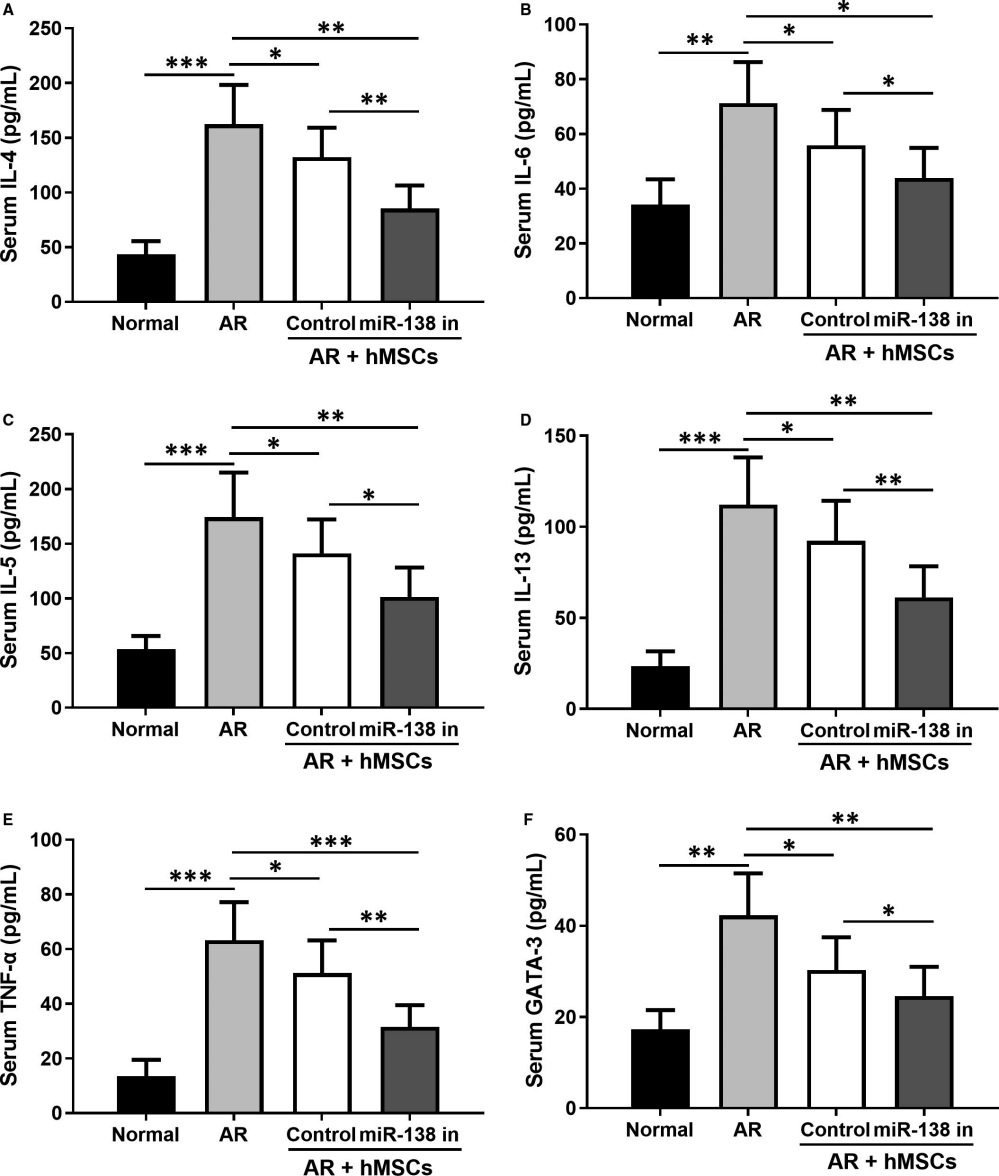
miR‐138‐5p inhibition modified hMSCs intranasal administration attenuated the inflammatory cytokines secretion. IL‐4 (A), IL‐6 (B), IL‐5 (C), IL‐13 (D), TNF‐α (E) and GATA‐3 (F) in the serum were measured by ELISA. N = 10

**FIGURE 7:**
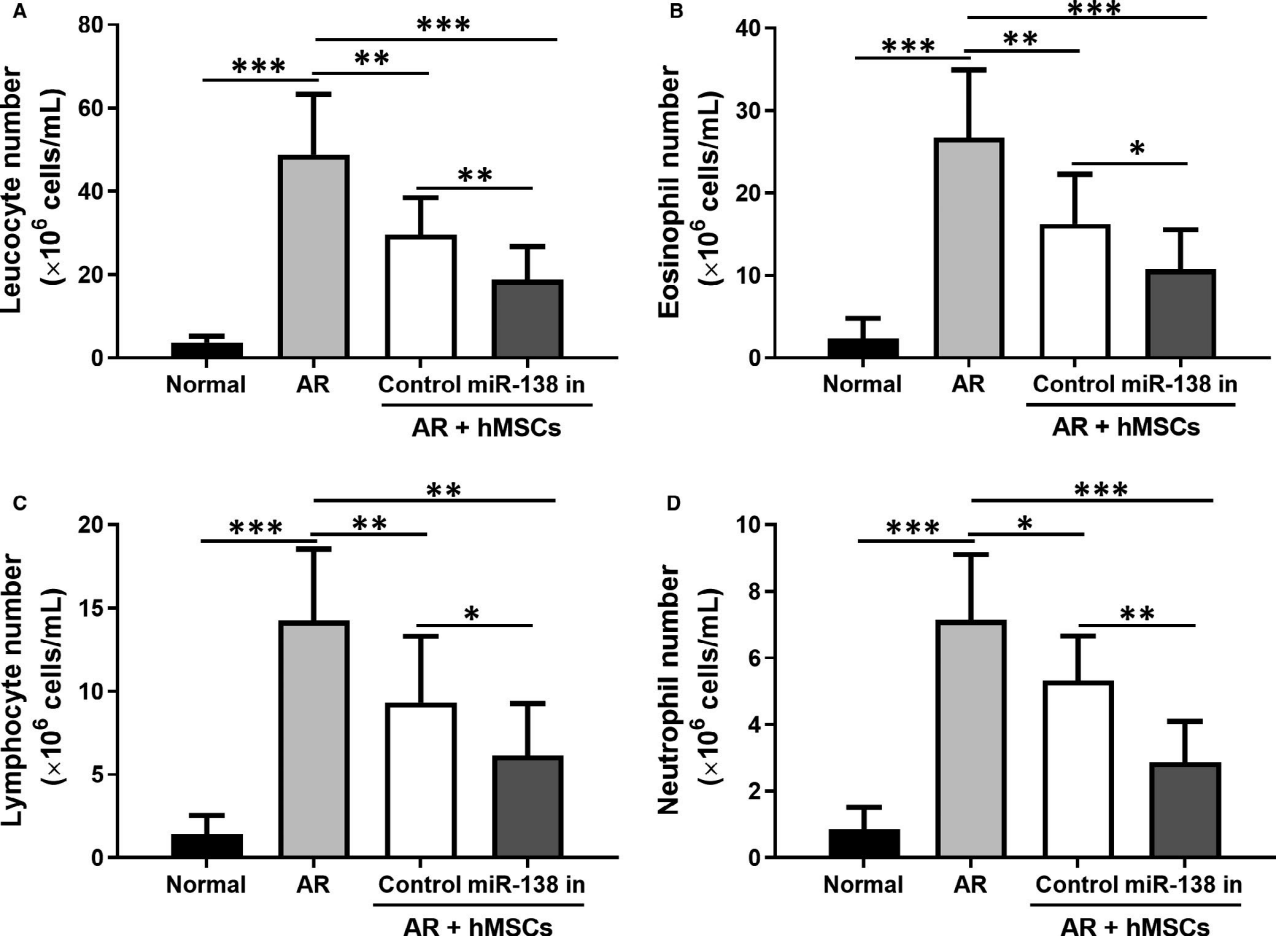
miR‐138‐5p inhibition modified hMSCs intranasal administration attenuates the number of leucocytes (A), eosinophils (B), lymphocytes (C) and neutrophils (D) in the nasal lavage fluid (NLF) of AR models. N = 10

We further analysed the expression of inflammatory factors in different groups and our results indicated that treatment with hMSCs with miR‐138‐5p inhibition was more effective than unmodified hMSC in reducing the expression of IL‐4 (Figure 6A), IL‐6 (Figure 6B), IL‐5 (Figure 6C), IL‐13 (Figure 6D), TNF‐α (Figure 6E) and GATA‐3 (Figure 6F). Furthermore, we also collected the NLF and counted the inflammatory cells after Wright's‐Giemsa staining under a light microscope. As shown in Figure 7, treatment with the unmodified hMSCs and engineered hMSCs led to significantly decreased numbers of leucocytes (Figure 7A), eosinophils (Figure 7B), lymphocytes (Figure 7C) and neutrophils (Figure 7D). The engineered hMSCs expectedly appeared to exert a greater effect in decreasing the numbers of inflammatory cells in the NLF.

### 3.8 Treatment with hMSCs with miR‐138‐5p inhibition activates *SIRT1* and inhibits the HMGB1/TLR4 pathway

**FIGURE 8:**
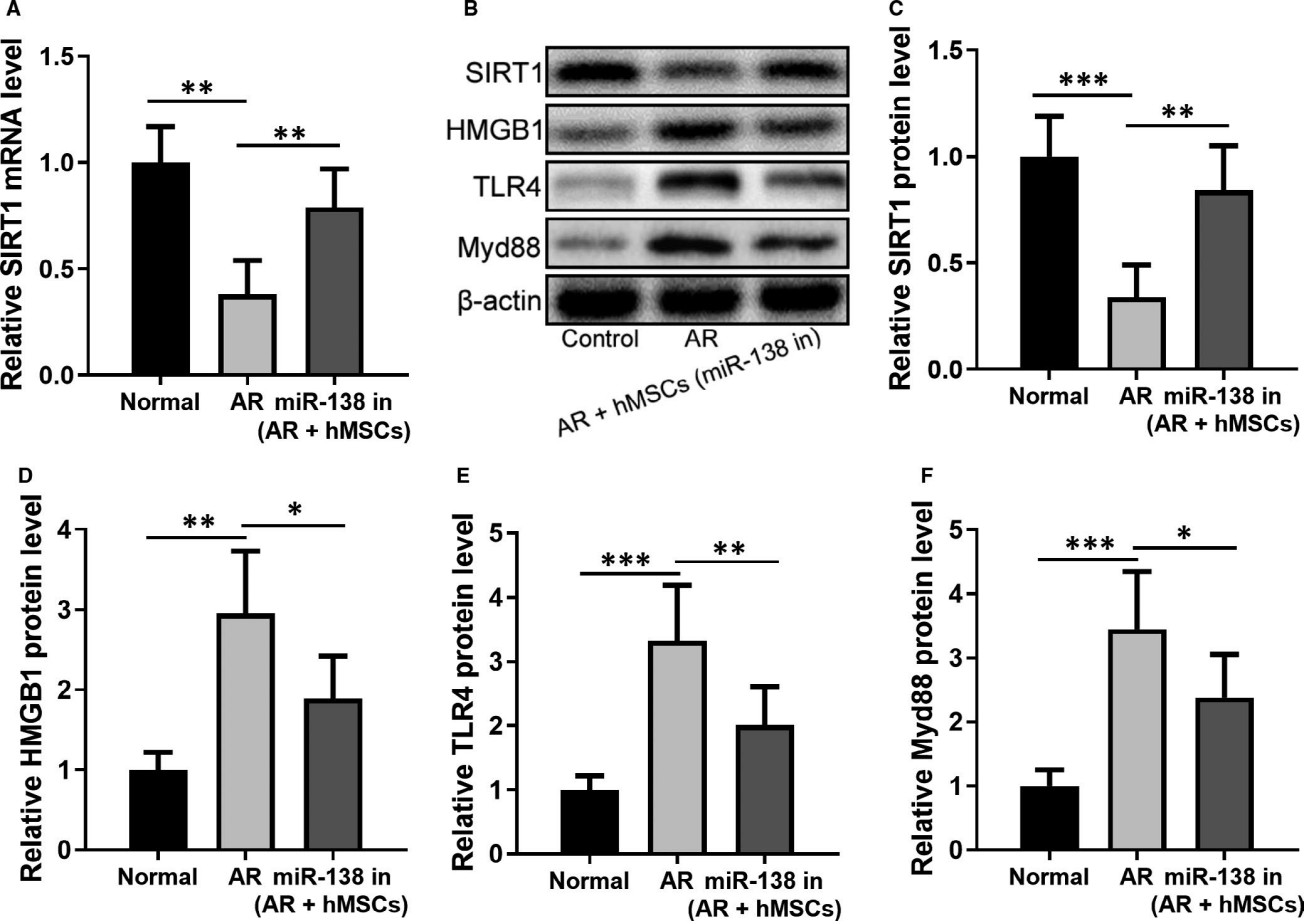
miR‐138‐5p inhibition modified hMSCs intranasal administration activated *SIRT1* and inhibited HMGB1/TLR4 pathway. qRT‐PCR was used to measure the mRNA expressions of *SIRT1* (A) in nasal mucosa from indicated groups. (B) Western blotting analysis of the protein expressions of SIRT1, HMGB1, TLR4 and Myd88 in nasal mucosa from indicated groups. β‐actin served as the loading control and expression levels were normalized to NC group (C‐F). N = 8

We next examined the effects of the miR‐138‐5p inhibitor engineered hMSCs in regulating *SIRT1* and HMGB1/TLR4 pathway in vivo. As shown in Figure 8A‐C, SIRT1 expression in mRNA and protein levels was markedly restored in the nasal mucosa tissue of AR mice (*P* < .01, compared to untreated AR rats, which showed a significant reduction of SIRT1). In the meantime, the activation of HMGB1/TLR4 pathway in AR mice was remarkably attenuated following the treatment of miR‐138‐5p inhibitor engineered hMSCs (Figure 8D‐F).

## 4 DISCUSSIONS

To date, current treatments for AR are limited to intranasal corticosteroids, antileukotrienes and antihistamines, which are merely symptom‐alleviating drugs and do not directly inhibit the allergic reactions. Although uncommon, the side effects, such as headache, throat irritation and nasal dryness, compromise the clinical utilities of these drugs.^24^ Hence, in this study, we aimed to develop a safer and more effective treatment for AR.

The use of hMSCs is an established safe clinical treatment that has been applied in many human malignancies, including steroid resistant graft vs host disease,^25^ Crohn's disease,^26^ amyotrophic lateral sclerosis,^27^ multiple sclerosis,^28^ etc The protective roles of hMSCs have been reported in a number of airway diseases, including pulmonary fibrosis,^29^ acute lung injury,^30^ AR and asthma.^10^ This promising application of MSCs in airway diseases has been supported by the fact that systemically administered MSCs can localize to the injured sites of the lung. Intraperitoneally injected MSCs could migrate to the lungs of newborn rats, protecting against hypoxia‐induced lung damage.^31^ Given the fact that AR is characterized by chronic inflammation with unbalance between TH1‐ and TH2‐derived cytokines and eosinophilic infiltration, we propose that immunomodulation driven by MSCs can contribute to inflammation reduction in AR.

To enhance the therapeutic efficacies of hMSCs, we exploited a genetic engineering approach, that is, the transfection of miR‐138‐5p inhibitor, to upregulate *SIRT1* expression in hMSCs. This rationale was based on our previous work that *SIRT1* negatively regulated the HMGB1/TLR4 pathway,^4^ which could be beneficial to the alleviation of allergic symptoms in AR. The direct binding of miR‐138‐5p to *SIRT1* (Figure 1) makes it feasible to transfect miR‐138‐5p inhibitor to increase *SIRT1* expression, while preserving the hMSC lineage (Table S1). This strategy has been employed by several previous studies and has been shown to effectively translate to immunoregulation.^32^ We also showed that miR‐138‐5p‐inhibitor‐engineered hMSCs became less sensitive to stimulation by TNF‐α and IL‐6, characterized by the attenuated expression of caspase‐3, IL‐6, IL‐1β and TNF‐α (Figure 2), implicating an augmented immunoregulatory capacity.

In this study, OVA intraperitoneal injection (day 1, 7 and 14) followed by repeated intranasal booster sensitization was used to induce allergic inflammation in a mouse model. This is a well‐established allergy model with sneezing and eye/nasal rubbing symptoms similar to those in the clinical settings.^33^ In this model, treatment with miR‐138‐5p‐inhibitor‐engineered hMSCs demonstrated superior efficacy in reducing sneezing and nasal rubbing events compared to unmodified hMSCs. Furthermore, the reduction of histamine in the serum and NLF served as additional supporting evidence showing that miR‐138‐5p inhibitor transfection in hMSCs could suppress allergies in the OVA‐induced AR model.

We next investigated the levels of several immunoglobulin antibodies implicated in TH cell‐mediated B‐cell immune responses, including IgE, LTF4, IgG1 and IgG2a. The significant elevation of the serum and NLF levels of OVA‐specific IgE, LTF4, IgG1 and IgG2a in the AR group was prominently abrogated by the treatment with hMSCs and miR‐138‐5p inhibitor engineered hMSCs. Because of the reciprocal roles of TH1‐ and TH2‐associated cytokines,^34^ the levels of several interleukins and interferons in the serum were also tested. Our data indicated marked reduction of IL‐4, IL‐6, IL‐5, Il‐13, TNF‐α and GATA‐3 by hMSC treatment (Figure 6). Expectedly, hMSCs engineered with miR‐138‐5p inhibitor showed a more potent reduction in these factors. Synergistically, reductions in both OVA‐specific immunoglobulin antibodies, inflammatory interleukins and interferons, contribute to the alleviation of allergic symptoms in AR mice.

Finally, we confirmed that the miR‐138‐5p inhibitor‐engineered hMSCs were able to restore *SIRT1* expression, while suppressing the HMGB1/TLR4 pathway in the nasal mucosa tissues from AR mice. Myd88 is a signalling adapter used by TLR4 to regulate interleukin expression,^35^ and the simultaneous downregulation of Myd88 was consistent with the inhibited HMGB1/TLR4 pathway.

It should be noted that miR‐138‐5p is only one of the miRNAs that regulate SIRT1. MiR‐34a,,^36^ miR‐29c,^37^ miR‐195,^38^ etc have all been found to regulate SIRT1 and are potential therapeutic target in hMSC‐based therapy. Our study is focusing on miR‐138‐5p as a lead gene and this study may help to establish a new modality for treatment of AR. In addition, miR‐138‐5p has also been found to regulate a number of targets besides *SIRT1*, such as surviving,^39^ ZEB2^40^ and PDK1.^41^ Here, we focused on *SIRT1* because of its significant role in AR, and *SIRT1* is one of the most investigated targets of miR‐138‐5p. But investigation of other molecular targets altered by miR‐138‐5p in hMSCs is warranted to fully characterize this AR therapy based on miR‐138‐5p inhibitor‐engineered hMSCs. One reason we chose to use miR‐138‐5p/*SIRT1* in hMSC engineering is that miR‐138‐5p/*SIRT1* is a relatively mature set of targets, which could facilitate the translation of this new hMSC‐based therapy. Another limitation of our study is that here we focused on hMSCs as the main therapeutic modality and miR‐138‐5p was used to synergistically enhance the treatment outcome. Direct use of miR‐138‐5p inhibitor, which could potentially be used as a control in our study, was not explored. Evaluation of the use of miR‐138‐5p inhibitor to directly treat AR is necessary to comprehensively assess the benefit of the engineered hMSCs for treating AR. Furthermore, the hMSCs were administered using intranasal injection, which is potentially less translatable than systemic injection of hMSCs, and future studies on systemically administered hMSCs are warranted to facilitate clinical translation of this hMSC‐based therapy in AR.

To sum up, this study reports the engineering of hMSCs with miR‐138‐5p inhibitor to enhance their immunoregulatory effects against ARAS. Our results indicate that the engineered hMSCs demonstrate superior efficacy in alleviating allergic symptoms, reducing histamine and OVA‐specific immunoglobulin antibodies in the serum and NLF, as well as several inflammatory cytokines. These effects are likely mediated by the activation of *SIRT1* and inhibition of the HMGB1/TLR4 pathway. Findings in the current study provide a viable approach to promote the use of MSC‐based therapies against ARAS and other inflammatory diseases.

## CONFLICT OF INTEREST

The authors declare that there is no conflict of interests.

## AUTHOR CONTRIBUTIONS

**Huaping Tang:** Data curation (lead); Formal analysis (equal); Funding acquisition (equal); Writing – original draft (equal). **Xiaolei Han:** Data curation (lead); Validation (equal). **Tingtian Li:** Data curation (equal); Formal analysis (equal). **Yan Feng:** Data curation (supporting); Validation (equal). **Jie Sun:** Conceptualization (lead); Resources (lead); Supervision (lead); Writing – original draft (equal); Writing – review and editing (equal).

## Supporting information

Supplementary Material

Supplementary Material
