# Supplementary material for: Protective effect of miR‐138‐5p inhibition modified human mesenchymal stem cell on ovalbumin‐induced allergic rhinitis and asthma syndrome
Source: J Cell Mol Med. 2021 May 11;25(11):5038–49. doi: 10.1111/jcmm.16473 (PMC8178307; doi:10.1111/jcmm.16473)
Supplement: Supplementary file 1 — Supplementary Material [file JCMM-25-5038-s001.docx]

**Supplementary materials**


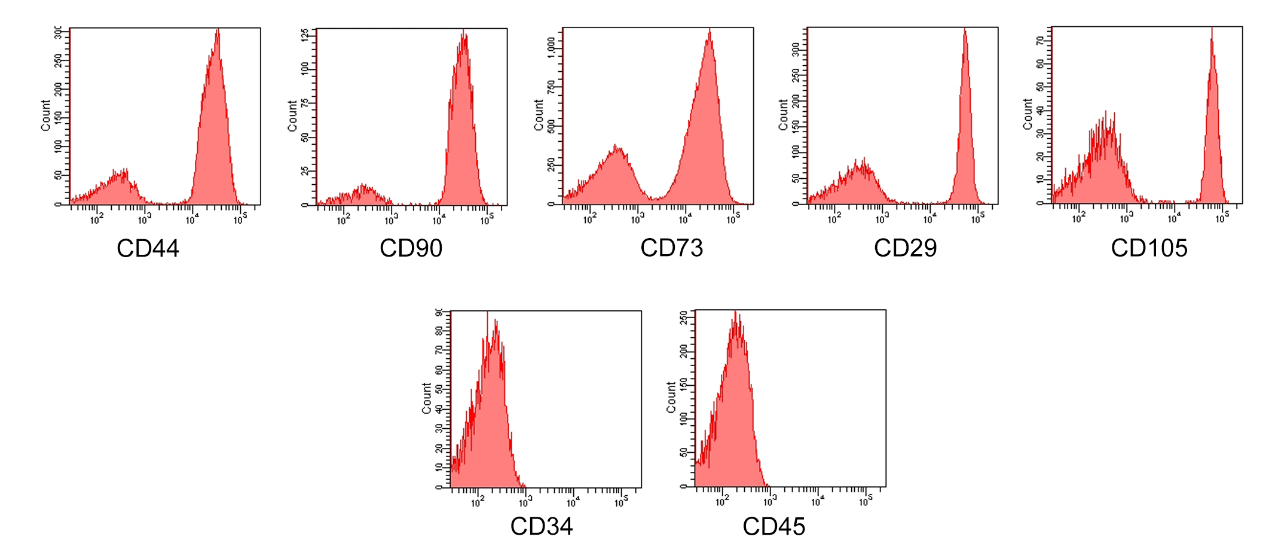


Figure S1. hMSCs were analyzed for CD 29, 34, 44, 90, 73, 105 and 45 expressions using by Fluorescence Activated Cell Sorting (FACS) with ﬂow cytometry at passage 4. The negative expressions of CD 34 and 45 and positive expressions of CD 29, 90, 73, 105 and 44.

Table S1. Analysis of cell surface marker expressions by ﬂow cytometry after transfection (%, mean ± SD).

| Groups (hMSCs) | n | CD29 | CD44 | CD34 | CD45 | CD90 | CD73 | CD105 |
| --- | --- | --- | --- | --- | --- | --- | --- | --- |
| Control | 4 | 98.64 ± 0.81 | 96.14 ± 1.51 | 0.43 ± 0.24 | 1.81 ± 0.39 | 94.61 ± 1.04 | 92.25 ± 2.42 | 96.26 ± 2.15 |
| miR-NC | 4 | 97.85 ± 0.92 | 95.21 ± 2.53 | 0.37 ± 0.11 | 2.14 ± 0.48 | 93.38 ± 1.13 | 93.47 ± 1.66 | 97.38 ± 1.79 |
| miR-138-5p inhibitor | 4 | 99.12 ± 0.83 | 94.15 ± 2.21 | 0.64 ± 0.37 | 1.94 ± 0.62 | 95.59 ± 1.63 | 94.13 ± 2.01 | 97.97 ± 2.73 |
| p value | - | 0.415 | 0.267 | 0.271 | 0.334 | 0.173 | 0.336 | 0.282 |

hMSCs among different groups were analyzed for CD 29, 34, 44, 90, 73, 105 and 45 expressions using by Fluorescence Activated Cell Sorting (FACS) with ﬂow cytometry at passage 4. The negative expressions of CD 34 and 45 and positive expressions of CD 29, 90, 73, 105 and 44 indicated a remaining mesenchymal stem cell lineage after miR-138-5p inhibitor transfection.

| Gene | Primer direction | Sequence  (5′--3′) |
| --- | --- | --- |
| hsa-miR-138-5p | Forward | GCTTAAGGCACGCGG |
|  | Reverse | GTGCAGGGTCCGAGG |
| SIRT1 (human) | Forward | AAAGGAATTGGTTCATTTATCAGAG |
|  | Reverse | TTGTGGTTTTTCTTCCACACA |
| U6 | Forward | TGCGGGTGCTCGCTTCGGCAGC |
|  | Reverse | GTGCAGGGTCCGAGGT |
| GAPDH (human) | Forward | ACAACTTTGGTATCG TGGAAGG |
|  | Reverse | GCCATCACGCCACAG TTTC |
| IL-6 (human) | Forward | GGT ACA TCC TCG ACG GCA TCT |
|  | Reverse | GTG CCT CTT TGC TGC TTT CAC |
| TNF-α (human) | Forward | ATC TTC TCG AAC CCC GAG TGA |
|  | Reverse | CGG TTC AGC CAC TGG AGC T |
| IL-1β (human) | Forward | CAACAGGCTGCTCTGGGATT |
|  | Reverse | CCTGGAAGGAGCACTTCATCT |
| caspase 3 (human) | Forward | CATGGAAGCGAATCAATGGACT |
|  | Reverse | CTGTACCAGACCGAGATGTCA |
| SIRT1 (mice) | Forward | CTGTTGACCGATGGACTCCT |
|  | Reverse | GCCACAGCGTCATATCATCC |
| β-actin (mice) | Forward | GTGGGAATGGGTCAGAAGGA |
|  | Reverse | TCATCTTTTCACGGTTGGCC |

Table S2. Oligonucleotide primer sequences for qRT-PCR.
