# Supplementary figures and images for: Protective effect of miR‐138‐5p inhibition modified human mesenchymal stem cell on ovalbumin‐induced allergic rhinitis and asthma syndrome
Source: J Cell Mol Med. 2021 May 11;25(11):5038–49. doi: 10.1111/jcmm.16473 (PMC8178307; doi:10.1111/jcmm.16473)

Supporting information  
Western blot uncropped films

Figure 1

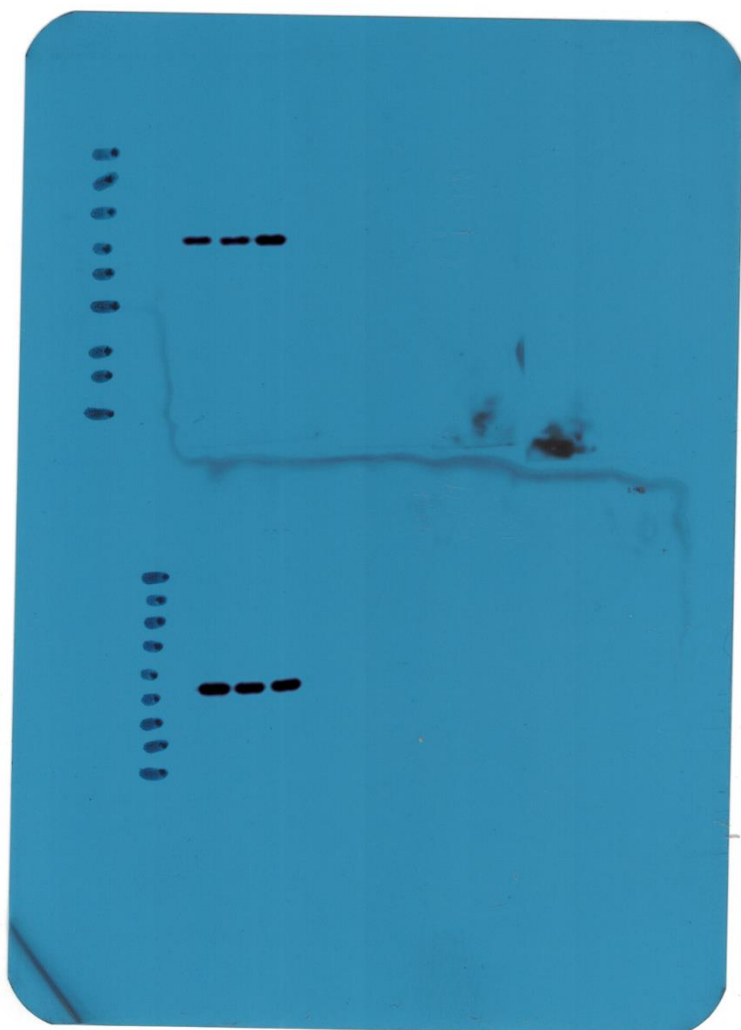

Figure 8

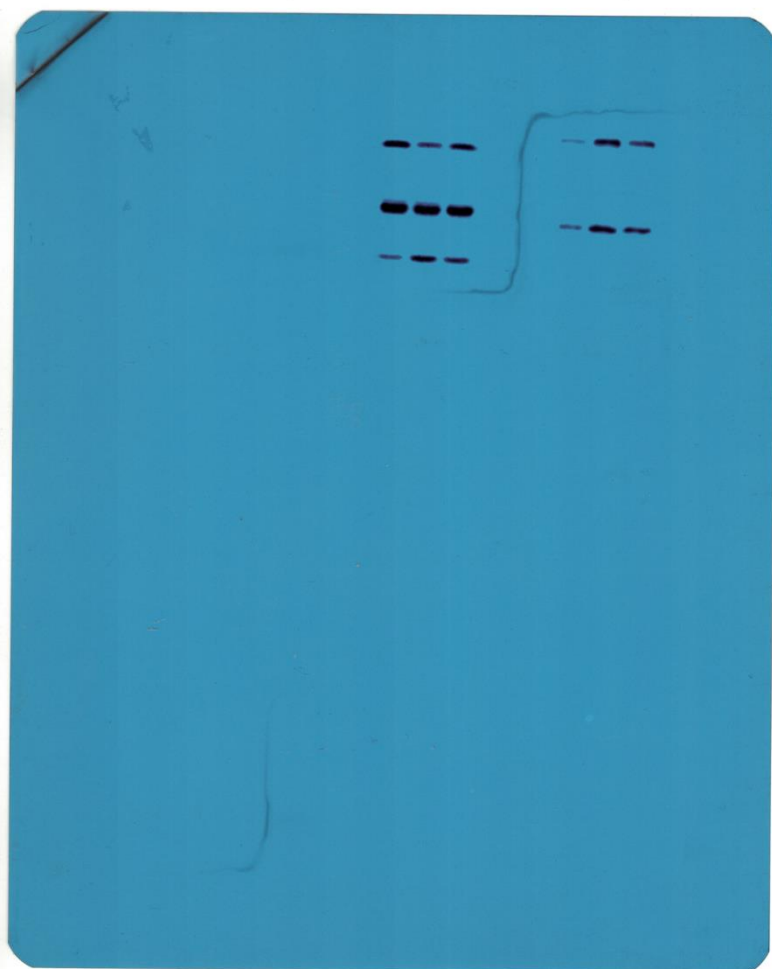

Supplement: Supplementary file 2 — Supplementary Material [file JCMM-25-5038-s002.pdf]
